# Human Umbilical Cord Mesenchymal Stem Cells to Treat Neuromyelitis Optica Spectrum Disorder (hUC–MSC–NMOSD): A Study Protocol for a Prospective, Multicenter, Randomized, Placebo-Controlled Clinical Trial

**DOI:** 10.3389/fneur.2022.860083

**Published:** 2022-04-25

**Authors:** Xiao-Ying Yao, Li Xie, Yu Cai, Ying Zhang, Ye Deng, Mei-Chun Gao, Yi-Shu Wang, Hui-Ming Xu, Jie Ding, Yi-Fan Wu, Nan Zhao, Ze Wang, Ya-Ying Song, Li-Ping Wang, Chong Xie, Ze-Zhi Li, Wen-Bin Wan, Yan Lin, Hai-Feng Jin, Kan Wang, Hui-Ying Qiu, Lei Zhuang, Yan Zhou, Yu-Yan Jin, Li-Ping Ni, Jia-Li Yan, Quan Guo, Jia-Hui Xue, Bi-Yun Qian, Yang-Tai Guan

**Affiliations:** ^1^Department of Neurology, Ren Ji Hospital, School of Medicine, Shanghai Jiao Tong University, Shanghai, China; ^2^Clinical Research Center, School of Medicine, Shanghai Jiao Tong University, Shanghai, China; ^3^State Key Laboratory of Oncogenes and Related Genes, Renji-Med-X Clinical Stem Cell Research Center, Renji Hospital, School of Medicine, Shanghai Jiao Tong University, Shanghai, China; ^4^Shanghai Clinical Research Promotion and Development Center, Shanghai Hospital Development Center, Shanghai, China

**Keywords:** neuromyelitis optica spectrum disorder (NMOSD), human umbilical cord mesenchymal stem cell (hUC-MSC), multicenter trial, randomized controlled trial, study protocol

## Abstract

**Background:**

Neuromyelitis Optica spectrum disorder (NMOSD) is severe relapsing and disabling autoimmune disease of the central nervous system. Its optimal first-line treatment to reduce relapse rate and ameliorate neurological disability remains unclear. We will conduct a prospective, multicenter, randomized, placebo-controlled clinical trial to study the safety and effectiveness of human umbilical cord mesenchymal stem cells (hUC–MSCs) in treating NMOSD.

**Methods:**

The trial is planned to recruit 430 AQP4-IgG seropositive NMOSD patients. It consists of three consecutive stages. The first stage will be carried out in the leading center only and aims to evaluate the safety of hUC—MSCs. Patients will be treated with three different doses of hUC–MSCs: 1, 2, or 5 × 10^6^ MSC/kg·weight for the low-, medium-, and high-dose group, respectively. The second and third stages will be carried out in six centers. The second stage aims to find the optimal dosage. Patients will be 1:1:1:1 randomized into the low-, medium-, high-dose group and the controlled group. The third stage aims to evaluate the effectiveness. Patients will be 1:1 randomized into the optimal dose and the controlled group. The primary endpoint is the first recurrent time and secondary endpoints are the recurrent times, EDSS scores, MRI lesion numbers, OSIS scores, Hauser walking index, and SF-36 scores. Endpoint events and side effects will be evaluated every 3 months for 2 years.

**Discussion:**

Although hUC–MSC has shown promising treatment effects of NMOSD in preclinical studies, there is still a lack of well-designed clinical trials to evaluate the safety and effectiveness of hUC–MSC among NMOSD patients. As far as we know, this trial will be the first one to systematically demonstrate the clinical safety and efficacy of hUC–MSC in treating NMOSD and might be able to determine the optimal dose of hUC–MSC for NMOSD patients.

**Trial registration:**

The study was registered with the Chinese Clinical Trial Registry (CHICTR.org.cn) on 2 March 2016 (registration No. ChiCTR-INR-16008037), and the revised trial protocol (Protocol version 1.2.1) was released on 16 March 2020.

## Introduction

Neuromyelitis Optica spectrum disorder (NMOSD) is a severe disabling inflammatory autoimmune disease of the central nervous system (CNS) featured with recurrent relapses of optic neuritis and longitudinally extensive transverse myelitis (1). Autoantibodies against the water channel protein aquaporin-4 (AQP4) are the diagnostic markers of the disorder and more than two-thirds of patients meeting clinical criteria for NMOSD are AQP4-IgG seropositive (2, 3). Frequent relapses are associated with a stepwise accumulation of neurological disability. Therefore, relapse prevention is particularly important to reduce the risk of a systemic disability over time (4). Azathioprine and mycophenolate mofetil are the most commonly used therapies for patients with NMOSD (5, 6). Recently, some randomized clinical trials show that the use of B-cell depletion (rituximab, inebilizumab), interleukin-6 signaling blockade (tocilizumab, satralizumab), and complement inhibition (eculizumab) can reduce the risk of relapses in NMOSD (7–11). However, still, some patients who have had these therapies have relapsed. So far, the optimal first-line treatment to reduce relapse rate remains unclear. Furthermore, no drug has been proved to improve neurological disability in NMOSD patients.

Stem cells are a group of cells that are immortal and have unlimited renewal abilities. The types of stem cells usually used in the registered clinical trials include multipotent stem cells, hematopoietic stem cells, and mesenchymal stem cells. There are some pilot trials and cohort studies assessing the therapeutic effect of stem cell transplantation in NMOSD patients (12–14). Recently, autologous nonmyeloablative hematopoietic stem cell transplantation showed to be effective on prolonged drug-free remission with AQP4-IgG seroconversion to negative (12).

Mesenchymal stem cells (MSCs) are stromal precursor cells residing in many tissues, including bone marrow, umbilical cord (UC), fetal liver, and adipose tissue (15–17). MSCs are considered to be multipotent and have anti-inflammatory, immune regulation, and paracrine effects, and also regenerative properties (18). MSCs derived from the human umbilical cord (hUC–MSCs) are more primitive, and possess multiple advantages including ethical agreeableness, a less-invasive procedure for isolation, low immunogenicity, high-proliferation capacity, and multi-lineage differentiation capability (19, 20). Therefore, hUC–MSCs are a promising candidate for cell-based therapy.

Treatment with MSC improves the course of the preclinical model of multiple sclerosis, experimental autoimmune encephalomyelitis (EAE) when administered at the early stages. In EAE, MSC has a profound anti-inflammatory and immune-modulating effect, but they also exhibit neuroprotective features and foster remyelination endogenous neurogenesis with scarce evidence of differentiation in neural cells (21–23). A pilot clinical trial showed that infusion of autologous MSC derived from bone marrow is safe, can reduce the relapse frequency, and mitigates neurological disability with the recovery of neural structures in the optic nerve and spinal cord in NMOSD patients (24).

Human umbilical cord–MSCs can regulate the immune response, promote tissue repair, and increase regeneration both *in vitro* and *in vivo* (25). Therefore, we suggest that hUC–MSCs can prevent relapses and ameliorate disability in NMOSD. However, as far as we know, there are very few reports on the use of hUC–MSCs in NMOSD patients. Here, we present the protocol of Human Umbilical Cord Mesenchymal Stem Cells to Treat Neuromyelitis Optica Spectrum Disorder (hUC–MSC–NMOSD), a prospective, multicenter, randomized, and placebo-controlled clinical trial. The main aims of the study are to verify that the hUC–MSCs can prolong the relapse interval, reduce the relapse times and ameliorate the neurological disability in NMOSD patients.

## Methods and Analysis

### Setting and Participants

The study will be conducted in six clinical centers in Shanghai, China. The leading center is Shanghai Ren Ji Hospital, which is one of the main teaching hospitals of the Shanghai Jiao Tong University School of medicine. Ren Ji Hospital is also one of the biggest clinical centers of neuroimmunological disorders in China. The five branch centers are Shanghai Rui Jin Hospital, Shanghai Xin Hua Hospital, Shanghai General Hospital, Shanghai Sixth People's Hospital, and Shanghai Ninth People's Hospital. In this study protocol, only the patients who fulfilled the diagnostic criteria of NMOSD established by Wingerchuk et al. (26) will be included.

The study protocol has been approved by the Ethics Committees of Ren Ji Hospital (2016-071K).

Patients will be recruited from those neuro-immunological centers of Neurology departments in China. Eligible patients will be invited to participate. All the participants will receive adequate information about the nature, purpose, possible risk, and benefits of the trial, and about alternative therapeutic choices. A signed written informed consent form will be obtained from all the participants before enrollment.

The clinical study will be conducted in accordance with all the national regulations and complies with the principles of the World Medical Association Declaration of Helsinki—Ethical Principles for Medical Research Involving Human Subjects, with all its amendments, and also with the principles of Good Clinical Practice.

The clinical information collected including age, sex, past medical history, NMOSD attack time and frequency, corrected visual acuity by standard logarithmic visual acuity chart, Expanded Disability Status Scale (EDSS) score, Opticospinal Impairment Scale (OSIS) score, Hauser walking index, quality of life short Form-36 (SF-36) score, serum status of anti-AQP4 antibody (tested in a cell-based assay), laboratory tests as listed in Table 1, electrocardiogram, abdominal ultrasound, chest X-ray or computed tomography, optical coherence tomography, number of brain and spinal cord lesions on MR images, immunomodulatory drugs used in the past 6 months, etc.

**Table 1:** Schedule of activities of human umbilical cord mesenchymal stem cells (hUC–MSCs) to treat neuromyelitis optica spectrum disorder (hUC–MSC–NMOSD) study.

**Follow-up Period**	**V0** **(Screening)**	**V1** **(Baseline)**	**V2**	**V3**	**V4**	**V5**	**V6**	**V7**	**V8**	**V9**
Time	**−30 ~ −1d**	**0**	**3M ± 7d**	**6M ± 7d**	**9M ± 7d**	**12M ± 7d**	**15M ± 7d**	**18M ± 7d**	**21M ± 7d**	**24M ± 7d**
General characteristics	√									
Inclusion and exclusion criteria	√									
Informed consent	√									
Physical examination	√		√	√	√	√	√	√	√	√
EDSS score	√		√	√	√	√	√	√	√	√
OSIS score	√		√	√	√	√	√	√	√	√
Hauser walking index	√		√	√	√	√	√	√	√	√
SF-36 score	√		√	√	√	√	√	√	√	√
Exploratory endpoints associated tests	√		√	√	√	√	√	√	√	√
Complete blood counts	√		√	√	√	√	√	√	√	√
Urinalysis	√		√	√	√	√	√	√	√	√
Coagulation function	√		√	√	√	√	√	√	√	√
Liver and kidney function	√		√	√	√	√	√	√	√	√
Serum glucose	√		√	√	√	√	√	√	√	√
Serum electrolyte	√		√	√	√	√	√	√	√	√
Thyroid function	√									
Urine HCG	√		√	√	√	√	√	√	√	√
Tests for infective diseases	√				√					√
Tumor markers	√		√	√	√	√	√	√	√	√
EKG	√		√	√	√	√	√	√	√	√
Abdominal ultrasound.	√		√	√	√	√	√	√	√	√
Chest X-ray or CT	√		√	√	√	√	√	√	√	√
MRI (head)	√					√				√
MRI (spinal cord)	√					√				√
MRI (optical nerve)	√					√				√
OCT	√					√				√
Primary treatment	√	√	√	√	√	√	√	√	√	√
hUC-MSC or placebo treatment		√	√	√	√					
Record of AE		√	√	√	√	√	√	√	√	√
Record of combined medications	√		√	√	√	√	√	√	√	√

### Study Design

This is a prospective study for an open-labeled, prospective, multicenter, randomized, placebo-controlled clinical trial. The estimated study period is from March 2019 to March 2025. The NMOSD patients in the remission phase will be randomized into two groups: the treatment group (hUC–MSC infusion plus regular treatment) and the controlled group (stem cell base solution infusion plus regular treatment). All the patients will be treated and followed up for 2 years.

The whole clinical trial includes three consecutive stages.

**Figure 1:**
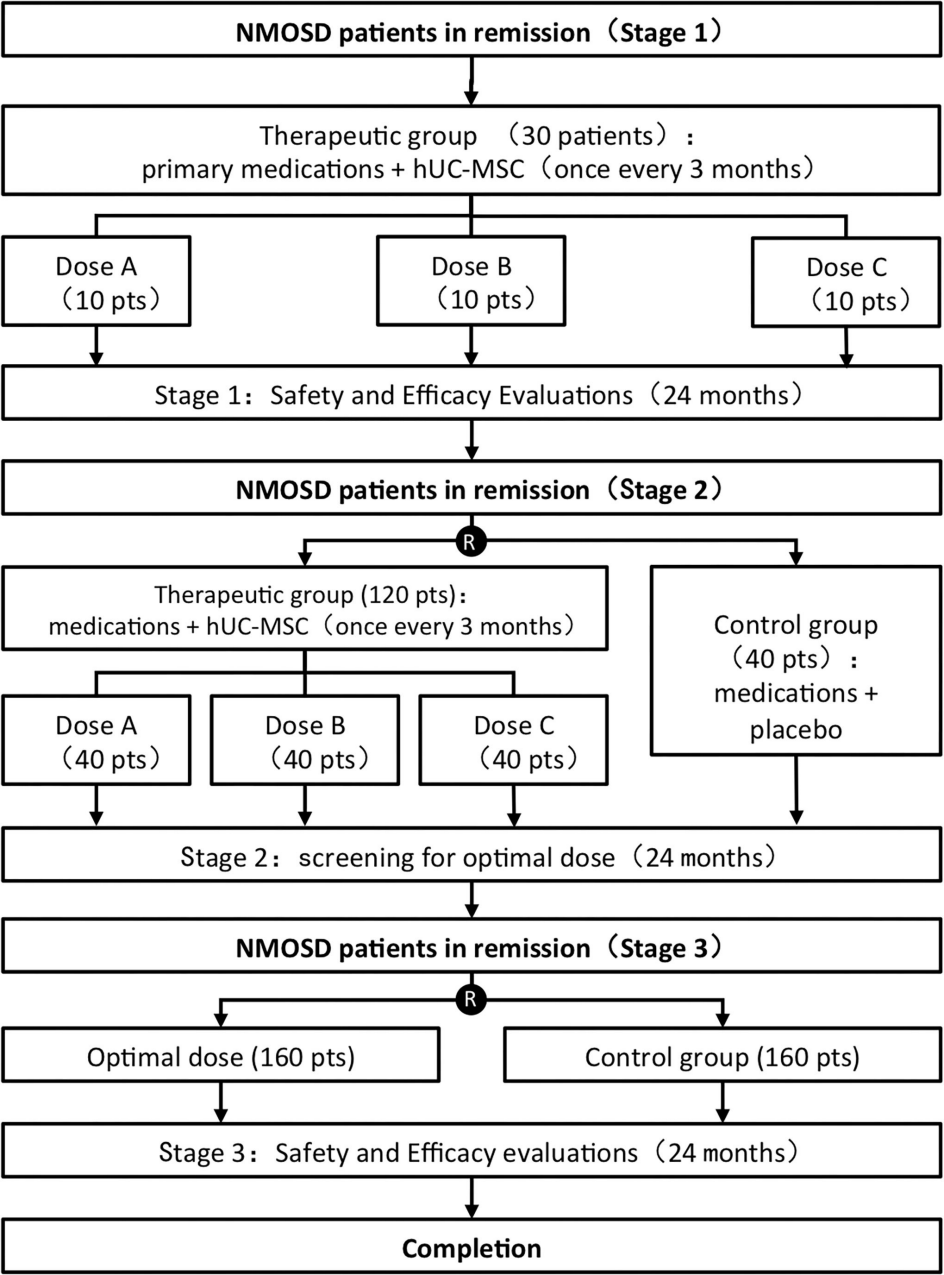
Study flow chart of human umbilical cord mesenchymal stem cells to treat neuromyelitis optica spectrum disorder (hUC-MSC-NMOSD) study.

The first stage of the trial, which will last 24 months, is an open-labeled study and will be carried out in the leading center only. The first stage aims to evaluate the safety of the infusion of hUC–MSC in NMOSD patients. Patients (*n* = 30) will be treated with three different doses of hUC–MSC. According to the process of enrollment, the first to the tenth participant is in the low-dose group (*n* = 10, 1 × 10^6^ MSC / kg·weight); the eleventh to the twentieth participant is in the medium-dose group (*n* = 10, 2 × 10^6^ MSC / kg·weight); the twenty-first to the thirtieth participant are in the high-dose group (*n* = 10, 5 × 10^6^ MSC / kg·weight). The study starts from the low dose. Only when the lower dose group ends the observation period of 3 months, the study will proceed to a higher dose group. In case of unacceptable toxic reaction occurs in the dose escalation, the study will be ended immediately. In relation to safety considerations, before enrolling the patients in the low-dose group, we will give a single medium dose of hUC–MSC to one patient and follow-up the therapy for one year. The outcome of this dosage will be used only for safety evaluation, not to evaluate the therapy's effectiveness.The second and third stages of the study protocol are part of the multi-center double-blinded randomized clinical trial. The second stage, which aims to screen out the optimal dosage, will last for 24 months and will be carried out in six centers (one leading center and five branch centers). Patients (*n* = 160) will be randomized into four groups: the low-, medium-, high-dose groups, and the controlled group (*n* = 40, respectively). In the second stage, we were seeking to determine the ‘optimal biological dose.’ The dose that allows reaching the optimal efficacy-toxicity trade-off is further selected and recommended to stage 3. The third stage, which aims to evaluate the effectiveness, will last for 24 months and will be developed in six centers. Patients (*n* = 320) will be randomized into two groups: the optimal dose group (*n* = 160) and the controlled group (*n* = 160). The third stage aims to evaluate the effectiveness and safety of hUC–MSC for NMOSD patients. The study flow chart is shown in Figure 1.

### Inclusion Criteria

1) 18–75 years of age;
2) Met the diagnostic criteria of NMOSD (established by the International Panel for NMOSD in 2015) (26), and serum AQP4-antibody-positive;
3) In the remission phase, defined as no new or worsening of neurological symptoms and signs in the last 30 days;
4) EDSS (Expanded Disability Status Scale) score ≤ 8;
5) Taking azathioprine or mycophenolate mofetil as ongoing treatment for NMOSD;
6) The results of the hepatic and renal function tests, complete blood count, and urinalysis are unremarkable;
7) The coagulation tests are unremarkable;
8) Be able to end the follow-up period;
9) Signed the informed consent (by patients or their families).

### Exclusion Criteria

1) Patients with language dysfunction, disturbance of consciousness, or unstable vital signs;
2) With thyroid dysfunction or other endocrine dysfunction which cannot be controlled by treatment;
3) With severe psychiatric disorders, thus unable to cooperate during the study;
4) With severe hepatic or renal disorders: the results of hepatic function >2 upper limits; the creatinine clearance rate calculated by Cockcroft–Gault equation <60 ml/min; or with severe systematic wasting diseases;
5) Scoring complete blood count test, white blood cell count test <3.0 × 10^9^/L or >12 × 10^9^/L, or hemoglobin level <80% lower limits, or platelet counts <100 × 10^9^/L;
6) Scoring coagulation tests, one or multiple indicators (TT, APTT, PT, FIB, TDP, and DD) >2 upper limits;
7) Gastrointestinal bleeding or other severe bleeding disorders in the last month;
8) Had allergic history to the study-related drugs;
9) Had major surgeries in the last week;
10) Pregnant or breastfeeding;
11) With a history of hepatitis B, hepatitis C, HIV infection, syphilis, or malignancies;
12) With other rheumatic diseases (such as Sjogren's syndrome, systematic lupus erythematosis), who need to use other immunodepressants other than azathioprine or mycophenolate mofetil;
13) With other situations that may affect the patient's engagement in the study.

### Withdrawal Criteria

Withdrawal criteria are as follows: (1) patient withdraws consent; (2) newly find misdiagnosis at baseline; (3) patients are lost to follow-up; (4) investigator's decision to withdraw the subject.

For any subject withdrawal, the reasons for withdrawal will be documented.

### Intervention

#### The Isolation and Culture of hUC–MSCs

The hUC–MSCs are isolated from Wharton's jelly (WJ) of the umbilical cord (UC). The parents who donated signed the informed consent. After aseptically collecting the samples of UC, scrape the WJ away from the blood vessels and inner epithelium of the subamnion. Then WJ is mechanically cut into small pieces of no more than a few millimeters in length. The pieces were transferred to culture flasks, which contained Dulbecco's Modified Eagle Medium-F12 (DMEM-F12) and 10% FBS (Fetal Bovine Serum), then it was incubated in a humidified atmosphere containing 5% CO2 at 37°C. The cells migrate from the explant to medium margins.

#### The Transportation and Storage of hUC–MSCs

The hUC–MSCs will be immersed in liquid nitrogen, carried in a portable liquid nitrogen container, and will be transported to the research centers by a cold chain logistic company. There will be a real-time recording of the temperature inside the liquid nitrogen container. The hUC–MSCs will be stored in liquid nitrogen until resuscitation and infusion.

#### Resuscitation of hUC–MSCs

A thermostat water bath will be preheated to 37°C. A metal clip will be precooled in liquid nitrogen and then the freezing bags of MSCs will be extracted. The freezing bags will be quickly dropped into the water bath and swayed back and forth gently until totally melting (time duration: 2 min).

#### Inspections of hUC–MSCs

The subjects can be infused hUC–MSCs only after the sample tests meets the following requirements:

(1) Total cell amount 5 × 10^7^ ± 10% per package; cell viability over 85%;
(2) Bacterial endotoxin test negative; gram-staining negative.

#### Transfusion of hUC–MSCs

Subjects in the intervention groups will receive four hUC–MSC infusions every 3 months besides the primary drugs. The dosages are 1, 2, or 5 × 10^6^ MSCs per kg of body weight. The hUC–MSCs will be infused *via* the subjects' peripheral veins. The drip rate is 30 drops per min in the first 15 min of infusion and 45 drops per min in the next 15 min. If there are no adverse effects, the drip rate can be gradually increased, but never exceed 70 drops per min. The time of infusion should last no more than 1 h. After the infusion of MSCs, subjects will be infused with 500 ml normal saline.

Subjects in the control group will receive four stem cell base solution infusions (the same packaging bag as hUC–MSCs) every 3 months besides the primary drugs.

#### Participant Safety Protection Measures

(1) Subjects will be admitted to the hospital when receiving the infusion;
(2) Intravenous infusion of dexamethasone of 5 mg will be given to subjects 15 min before the infusion of hUC–MSCs;
(3) Subjects will have electrocardiogram monitoring during the whole process of infusion;
(4) Subjects will be discharged after a 2–4 days observation period after the infusion.

### Group Allocation, Randomization, and Blinding

The first phase of the trial is an open-label, dose-escalation study that will be performed in a leading center, the Shanghai Ren Ji Hospital. For stages 2 and 3, allocation sequences were generated by an independent statistician by permuted blocks randomization method (block sizes of four or eight). The conceal of allocation sequences were performed by using an integrated web response system (IWRS). The research physician will enroll participants and then assign participants to interventions according to allocation results from IWRS. In stage 2, there are four arms, which are the low dose group (1 × 10^6^ MSC / kg·weight), the medium dose group (2 × 10^6^ MSC / kg·weight), the high dose group (5 × 10^6^ MSC / kg·weight), and the control group (stem cell base solution). Patients will be assigned randomly in a 1:1:1:1 ratio to hUC–MSC groups and control group, respectively. In stage 3, there are two treatment arms. Patients will be assigned randomly in a 1:1 ratio to the hUC–MSC group and control group, respectively. To avoid ethical problems, we designed an add-on trial. Patients in the placebo-controlled group will receive a placebo while they already receive an established regular treatment (azathioprine or mycophenolate mofetil). Therefore, the allocation ratio of 1:1 was based on optimal statistical power and add-on trial design. In stages 2 and 3, all the participants, investigators, and statistician are blinded to group allocation until the end of the study and data analysis. Only pharmacists who are in charge of preparing study drugs are not blinded. Double-blinding will be performed *via* a centralized randomization system. Subjects who leave the study after random allocation will not be replaced.

### Sample Size Estimation

Based on previous epidemiology studies on NMOSD among Asians, the replacing rate for the patients in the first year of the onset event is estimated at 55% (27). In this study, we expected that the relapsing rate could be reduced to 40% in the MSC group, according to our preliminary data. In the first stage of an exploratory study, each dosage cohort will include 10 patients to meet the safety evaluation goal, which are 30 patients in total. In the adaptive design stages 2 and 3, a total sample size of 400 patients would have 80% power to detect 15% relapsing rate reduction at a two-sided 5% significant level, with a 20% expected loss to follow-up rate. Therefore, we expected to enroll a total number of 430 subjects in this study. The sample size of this study was determined according to the stage of trial by R software (Version 4.0.3).

### Discontinuation of Study Intervention

Unexpected discontinuation of study intervention criteria is (1) deterioration of symptoms leading to MSC infusion intolerance; (2) occurrence of adverse events or abnormal laboratory test results leading to MSC infusion intolerance; (3) individual wishes of the subjects; (4) any reasonable situations that require the clinical trial to be halted; (5) researchers' decisions to halt the clinical trial.

Expulsion criteria are (1) non-compliance to the study protocol; (2) other reasons.

### Primary and Secondary Efficacy Endpoints

The primary study endpoint is defined as the first time of recurrence. The recurrence should meet all the following four requirements: occurrence of new symptoms or deterioration of original symptoms; symptoms lasting for more than 24 h; more than 30 days from the last recurrence; no other reasons to explain the recurrence.

Secondary endpoints include the times of recurrence, EDSS scores, MRI lesion numbers, OSIS (Opticospinal Impairment Scale) scores, Hauser walking index, and SF-36 (quality of life short Form-36) scores. MRI lesion numbers refer to the total numbers of the lesions on T2-weighted images of the brain, the cervical, and the thoracic spinal cord, respectively. The SF-36 questionnaire is one of the most widely used qualities of life measures and it has been translated and validated in China (28). It has eight multi-item domains including physical functioning, social function, role limitations related to physical problems, role limitations related to emotional problems, mental health, vitality, bodily pain, and general health perceptions. SF-36 results are presented in two categories of physical component summary (PCS) and mental component summary (MCS). EDSS scores, OSIS scores, Hauser walking index, and SF-36 scores will be assessed every 3 months.

Exploratory endpoints include: serum lymphocyte subsets (T helper cells, T suppressor cells, Natural Killer cells, and B lymphocytes), cytokines (Interleukin-2,4,6,10,17A, TNF-α, and IFN-γ), complements, serum anti-AQP4 antibody titers, average retinal nerve fiber layer (RNFL) thickness, etc.

### Adverse Events

Adverse events (AEs) refer to any unwanted symptoms or signs in a clinical investigation subject and do not necessarily have a causal relationship with the applied intervention (29). All AEs will be coded according to the WHO's Adverse Reactions Terminology. At the end of the clinical trial, the intensity and relationship of any AEs with the study intervention will be identified.

If any severe AE (SAE) is encountered, even with extremely low incidence, it will be reported to the trial's principal investigator, the stem cell research ethics committee, and the state supervision agency within 24 h of its occurrence.

There are two types of side effects that need intensive monitoring, side effects during infusion and long-term side effects.

Side effects during infusion include allergic reactions (such as fever, tachycardia, dyspnea) and systematic complications (infection, embolism, and multiple organ failure);

While monitoring long-term side effects, the following items will be tested every 3 months: tumor markers (AFP, CEA, CA199, CA125, CA153, and CA242); complete blood count and urinalysis, liver and kidney function, blood glucose and electrolyte, coagulation tests, tests for infective diseases (HIV, RPR, hepatitis B, and C; tested at baseline, 9 months and 2 years of follow-up), urine HCG; electrocardiogram, chest X-ray or CT, and abdominal ultrasound.

### Follow-Up

Primary, secondary, exploratory endpoint events, and side effects will be evaluated in all participating subjects at baseline and every 3 months. Magnetic resonance scans and optical coherence tomography (OCT) will be evaluated at baseline, year 1, and year 2 of follow-up.

For safety consideration, during stage one of the trial, all the subjects will receive a telephone interview within 1 week after hUC–MSC infusion and then every 2 weeks until the second infusion. The telephone interview will include information on symptoms' change and side effects.

The study schedule is shown in Table 1.

### Data Management

All the study documents from all the centers will be considered highly confidential and will be stored in locked filing cabinets in a room with restricted access. All data will be translated into electronic format and stored in a database. Access to the database will be strongly restricted. The principal investigator and the biostatistician will be able to log into the data set and get full access to the information only upon permission from the head of this study. Data backups will be performed regularly by trial coordinators. If required, data transfer between centers will be encrypted, and any information capable of identifying individuals will be removed.

### Trial Quality Assurance

This study will be implemented according to high-quality standards and will be delivered in accordance with the present trial protocol, which will not be amended unless any SAE occurs during its implementation. All the research staff, including investigators, research assistants, and outcome evaluators, will be trained in advance to be able to competently administer all the items as per the protocol. Once the clinical trial begins, an independent trial inspector will visit each study site monthly and will be responsible for reviewing the following: overall research progress and integrity, adherence to the selection criteria for all the included subjects, compliance with the scheduled intervention for each participant, compliance with national regulations, and the handling of practical problems. Moreover, this trial inspector may occasionally provide suggestions to the principal investigator, who will make any final decisions about trial modifications, continuation, or termination.

### Statistical Analysis

The Kolmogorov–Smirnov Z test will be used for checking data normality. Categorical variables will be summarized as counts (percentage) and continuous variables as the means (standard error, SE) or medians (interquartile ranges, IQR), if not normally distributed. For the primary endpoint of the first time of recurrence, survival analysis will be conducted to analyze between-group differences. The Kaplan–Meier estimates for time of recurrence are presented for all groups. Hazard ratios for the comparison of between groups and 95% CIs were calculated with the Cox proportional-hazards model. Prespecified subgroup analyses of the primary outcome were stratified by, EDSS score at baseline, disease duration, age at randomization, the total number of previous relapses, number of relapses 2 years before randomization, and regular treatment. For secondary outcomes, statistical comparisons between the groups will be performed using Student's *t*-test or the Mann–Whitney U test for continuous variables, as well as the chi-square and Fisher's exact tests for categorical variables, as deemed appropriate. Correlations between continuous variables will be assessed by Pearson's correlation coefficient or Spearman's correlation coefficient. All missing data were not imputed in this study.

In this study, two analysis sets will be required. For the safety set (SS) analysis, all safety indicators will be obtained from those who have undergone at least one dose of hUC–MSC infusions. For the effectiveness analysis set, the intention to treat (ITT) analysis set will be used.

Lan–DeMets alpha-spending function with an O'Brien–Fleming boundary will be used to control the family-wise of type I error. Inverse normal combination test will be used to combine data from stages 2 and 3 in the final analysis. An independent Data and Safety Monitoring Board (DSMB) will review and evaluate the accumulated study data for participant safety, study conduct, and progress at each stage of the study. Only members of DSMB could have access to the data and results of the analysis. The Ethics Committee will be notified of any amendment to the study protocol. DSMB can make recommendations to terminate the study based on one of the following conditions: a significant difference in terms of effectiveness between groups; a significant risk-benefit ratio in one group; low degree of success in a reasonable period (such as poor compliance, low incidence of endpoint events). The advice of the DSMB meeting will be shared with the steering committee of the trial.

## Discussion

To the best of our knowledge, this study will be the first one to systematically demonstrate the clinical safety and efficacy of hUC–MSC in treating NMOSD, which leads to severe neurological disability in the young population. Several studies have reported preliminary results on the efficacy of hUC–MSC in treating different types of diseases, such as stroke, spinal cord injury, hereditary spinocerebellar ataxia, multiple system atrophy, multiple sclerosis, and other systemic diseases such as hematological diseases, immunological diseases, etc. (30–36). Toxicity is rarely reported in these studies. In addition, these studies have a small number of subjects, lack control, and are not well-designed. Therefore, there is a need for high-quality evidence on the efficacy of hUC–MSC transfusion.

Some studies with small sample sizes have reported the efficacy of stem cells in treating NMOSD, but they use invasive methodologies in performing stem cell transplantation (12–14). As far as we know, there was only one study using hUC–MSC to treat NMO till the present day. This study was published in 2012. In this study, hUC–MSCs transplantation was used to treat five NMO patients with a follow-up period of 18 months. Among the five cases, four showed therapeutic improvement after hUC–MSCs treatment. Both symptoms and signs improved and relapse frequencies were reduced (37).

The majority of previous studies only tested one dose of hUC–MSCs (36). Therefore, the optimal transfusion dose of hUC–MSC has not been determined till now. We use three different doses (1, 2, or 5 × 10^6^ MSC per kg of body weight) in stages 1 and 2 of the trial in order to determine the optimal dose of hUC–MSC in NMOSD patients, balancing both efficacy and safety.

The route of administration of hUC–MSCs also needs to be determined. The rationale for intrathecal management is the transportation of cells directly into the central nervous system (CNS). However, intrathecal injection of hUC–MSCs does not affect cytokine dissimilarity in peripheral blood (38). Besides, intrathecal administration in humans may lead to meningeal irritation, likely related to CNS inflammation, which can cause headache, seizures, acute encephalomyelitis, etc (39, 40). On the contrary, intravenous administration of hUC–MSCs is not invasive and has very mild self-limited adverse events (39, 40). Therefore, it should be considered as a preferred route.

This study is a well-designed protocol for a clinical trial, and it includes a variety of safety indicators, functional assessments, imaging evaluations, and humoral indicators. We believe that this study protocol will allow us to elucidate the therapeutic efficacy and safety concerns of hUC–MSC in treating AQP4-positive NMOSD and finally to determine the best dosage to be used.

## Conclusion

In summary, hUC–MSC holds great promise for an effective treatment of NMOSD.

The results of this study may represent a milestone and may be used for future revisions of NMOSD guidelines.

## Ethics Statement

The studies involving human participants were reviewed and approved by the Ethics Committees of Ren Ji Hospital. The patients/participants provided their written informed consent to participate in this study.

## Author Contributions

Y-TG, B-YQ, X-YY, LX, YC, YD, Z-ZL, CX, W-BW, and YZha: conception and design. Y-TG and B-YQ: administrative support. X-YY, M-CG, Y-FW, NZ, ZW, Y-YS, H-MX, Y-SW, JD, L-PW, KW, H-YQ, LZ, YZho, Y-YJ, L-PN, J-LY, QG, J-HX, YL, and H-FJ: provision of study materials or patients. X-YY, M-CG, Y-FW, NZ, ZW, Y-YS, L-PW, and JD: collection and assembly of data. X-YY and LX: data analysis and interpretation. All authors: manuscript writing and final approval of manuscript.

## Funding

This study was supported by the National Key Research and Development Project of Stem Cell and Translational Research by the Ministry of Science and Technology (Project No. 2020YFA0113100), National Natural Science Foundation of China (Project Nos. 81771295 and 81801211), and the Funding of Multicenter clinical research projects of School of Medicine, Shanghai Jiao Tong University (Project No. DLY201605).

## Conflict of Interest

The authors declare that the research was conducted in the absence of any commercial or financial relationships that could be construed as a potential conflict of interest.

## Publisher's Note

All claims expressed in this article are solely those of the authors and do not necessarily represent those of their affiliated organizations, or those of the publisher, the editors and the reviewers. Any product that may be evaluated in this article, or claim that may be made by its manufacturer, is not guaranteed or endorsed by the publisher.
